# Correspondence in relation to the case report "Capnography as an aid in localizing the phrenic nerve in brachial plexus surgery. Technical note." published in May issue of Journal of Brachial Plexus and Peripheral Nerve Injury

**DOI:** 10.1186/1749-7221-3-20

**Published:** 2008-10-22

**Authors:** Pradipta Bhakta

**Affiliations:** 1 Dept. of Anaesthesiology and Intensive Care, Sultan Qaboos University Hospital, Muscat, Oman

## Abstract

Comment on '**Capnography as an aid in localizing the phrenic nerve in brachial plexus surgery. Technical note' Bhagat H, Agarwal A, Sharma MS**

*Journal of Brachial Plexus and Peripheral Nerve Injury *2008, **3**:14 (22 May 2008)

## 

Dear Editors,

I want to thank the authors for this article explaining this innovative technique to identify phrenic nerve intraoperatively. This may be applied a good technique as replacement of currently available means. But after going through the article I found some doubts related to the actual correlation of diaphragmatic contraction with electrical stimulation of phrenic nerve.

Authors have used an intravenous based anesthesia for their cases without muscle relaxation. They have not mentioned anything about the dose of the drug used or monitoring the depth or adequacy of anesthesia. Nor they mentioned anything about intraoperative ventilatory technique during maintenance of anesthesia. From the pattern of the capnogram presented in the report, I can assume that probably a controlled ventilatory technique was used in all the cases [1]. Authors have assumed that notches in the alveolar plateau part (phase III) of capnogram were because of diaphragmatic contraction elicited by electrical stimulation. But there are several reasons of appearance of notch in phase III of capnogram namely curare cleft, hiccup, premature respiratory effort by the patient during mechanical ventilation etc [1-3]. Though curare cleft is out of question in these cases, but premature respiratory effort provoked by painful electrical stimulation in the scenario of inadequate anesthesia and analgesia should have been considered as a possibility [1-3]. It is very well known that any electrical stimulation above 1–2 mA is very painful [4]. That is why it is advised to start electrical stimulation with lowest possible current and to increase it until stimulation is obtained. Though some or most of these painful responses can be reduced or abolished by use of anesthesia, painful stimulation like this under inadequate anesthesia can manifest as hemodynamic imbalance as well as premature respiratory effort. Appearance of cleft in capnogram mentioned here is also similar to that seen in case of premature inspiratory effort [1]. This is specifically important when patient is kept on ventilator with or without muscle relaxation or anesthesia and analgesia are inadequate. There was no mention of hemodynamic response to electrical stimulation. This could have dictated us about rough guide of adequacy of anesthesia in absence of any specialize depth of anesthesia monitoring. That's why monitoring of anesthetic depth and mentioning of drug dose are important. Nothing was mentioned to rule out this possibility in the case report. Even nothing was mentioned about occurrence of hiccup, which is usually seen in case of phrenic nerve stimulation [5]. This is a possibility in case of electrical stimulation when muscle relaxant is not used.

They have also mentioned something about progressive reduction in ETCO_2 _level in subsequent capnogram tracings. But they have not given any valid explanation to the cause of this occurrence. Ventilator setting here is very important. They have not mentioned anything about the ventilator rate and tidal volume setting in their particular case in relation to capnogram recording. Hypocarbia can result from several causes. Most common of them is hyperventilation (iatrogenic or induced by patient due to inadequate anesthesia) [1]. Again premature respiratory effort due to painful electrical stimulus can lead to hyperventilation leading to reduction of end tidal CO_2_.

Thus I must admit here that this case report is a little bit inadequate in ruling out other possibilities of diaphragmatic contractions rather than elicited by electrical stimulation of phrenic nerve. Thus before accepting this method as a new and innovative technique to detect phrenic nerve in difficult surgical condition like this, this common possibility should be ruled out. I hope to see some valid explanation relating to my queries from the authors.

Thanking you-

Sincerely yours,

Pradipta Bhakta

## References

[B1] Moon RE Camporesi EM Miller RD Respiratory monitoring Miller's Anesthesia 2005 6 Philadelphia: Elsevier Churchill Livingstone 1455 462

[B2] Dorsch JA Dorsch SE editors Understanding anesthesia equipment 1994 3 Baltimore: Williams & Wilkins 581 596

[B3] Hensler T Dhamee MS Anesthesia machine malfunction simulating spontaneous respiratory effort J Clin Monit 1990 3 128 131 10.1007/BF02828289 2112591

[B4] Hadzic A Vloka JD Claudio RE Electrical nerve localization: effects of cutaneous electrode placement and duration of stimulus on motor response Anesthesiology 2004 3 1526 1530 10.1097/00000542-200406000-00027 15166574

[B5] Raj PP Andres JD Grossi P Raj PP Aids to localization of peripheral nerves Textbook of regional anesthesia 2003 New York: Churchill Livingstone 309

